# ESPO AND HEALTH SCIENCES SECTION SYMPOSIUM: BUILDING BRIDGES: LEVERAGING SOCIAL CONNECTIONS AND ENVIRONMENTS TO IMPROVE HEALTH IN LATER LIFE

**DOI:** 10.1093/geroni/igad104.1501

**Published:** 2023-12-21

**Authors:** Kyle Moored, Katherine Britt, Thomas Cudjoe

## Abstract

The COVID-19 pandemic has renewed focus on social connections as fundamental contributors to health across the lifespan. Social connections have been studied in numerous ways (e.g., social network size, daily social interactions, engagement in social activities) and may facilitate health via multiple, dynamic biopsychosocial mechanisms. In line with this year’s theme, we review novel insights from early-career scholars into how “building bridges” with others throughout life may promote mental and cognitive health in later life. Further, we emphasize how bridging disciplines (e.g., social and neurocognitive sciences) is vital to understanding how social environments may “get under the skin” to impact health. Presentations will span multiple social-ecological levels (community, institutional, interpersonal). Beginning at the community/institutional level, Dr. Moored will discuss how the quantity of neighborhood social destinations contributes to GPS measures of community mobility in older adults. Dr. Britt will then describe how religious participation, a specific social institutional activity, is associated with an inflammatory biomarker predictive of cognitive impairments. The remaining talks will then report on daily social experiences. Dr. Ng will highlight links between daily support exchanges and life satisfaction in older adults and how these relationships differ by marital status. Extending to the caregiving context, Dr. Puga will present on how role captivity and daily changes in social isolation contribute to mood symptoms in family caregivers of individuals living with dementia. Our Discussant, Dr. Cudjoe, will provide a critical review in the context of new directions for research on social connections and environments.

